# Linezolid versus daptomycin treatment for periprosthetic joint infections: a retrospective cohort study

**DOI:** 10.1186/s13018-019-1375-7

**Published:** 2019-10-24

**Authors:** Masahiro Sawada, Kenichi Oe, Masayuki Hirata, Hiroshi Kawamura, Narumi Ueda, Tomohisa Nakamura, Hirokazu Iida, Takanori Saito

**Affiliations:** Department of Orthopaedic Surgery, Kansai Medical University, 2-5-1 Shinmachi, Hirakata, Osaka 573-1010 Japan

**Keywords:** Linezolid, Daptomycin, Periprosthetic joint infection, Implant retention, Adverse event rates

## Abstract

**Background:**

Linezolid (LZD) and daptomycin (DAP) are predominantly used to target gram-positive pathogens; however, treatment effectiveness and adverse reactions for periprosthetic joint infections (PJIs) remain unknown. The aim of this study was to compare the effectiveness and adverse reactions of LZD and DAP for PJIs.

**Methods:**

This study retrospectively evaluated 82 patients between June 2009 and December 2017, to compare the effectiveness of LZD (group L, *n* = 39) and DAP (group D, *n* = 43) for treatment of PJIs harboring gram-positive microorganisms. Surgical options used with LZD or DAP therapy included implant retention, implant removal, and a shift to another appropriate antibiotic. Infection control was defined as not requiring implant removal after the final treatment.

**Results:**

Gram-positive pathogens were isolated from 72% of group L and 70% of group D patients, respectively. Whole infection control rates against gram-positive pathogens in groups L and D were 79% and 77%, respectively. Furthermore, infection control rates were 94% and 58% in group L and 75% and 80% in group D, without and with implant removal, respectively. Significantly higher clinical success rates and lower adverse event rates were observed in group D, including higher red blood cell and platelet counts and lower C-reactive protein (CRP) levels.

**Conclusions:**

Although the effectiveness of LZD and DAP was equivalent in terms of infection control rates for refractory PJIs with gram-positive pathogens, DAP therapy significantly decreased CRP levels and caused fewer adverse events than LZD treatment.

## Background

According to the Nordic Arthroplasty Register Association, the number of periprosthetic joint infections (PJIs) is increasing [1], and there are differences between PJIs and other infections with regard to the pathogenesis and treatment strategies. In PJIs, subclinical infections frequently occur by conventional diagnostic procedures because the bacteria subsist within biofilms on implant surfaces [2]. Therefore, implant removal is generally required for the treatment, and the probability of implant retention is limited [3]. Although the gold standard for managing PJIs includes both implant removal and thorough debridement, accompanied by an appropriate antibiotic therapy, there is no consensus for the treatment of PJIs regarding the choice of specific antibiotic therapy. Furthermore, the treatment of PJIs should target gram-positive pathogens because such organisms cause the majority of PJIs and antibiotic resistance is difficult to overcome [4–7].

Linezolid (LZD) and daptomycin (DAP), which are specific antibiotics used to treat gram-positive pathogens, are currently available as novel antibiotic therapies worldwide. LZD, the first oxazolidinone antimicrobial, inhibits bacterial protein synthesis by preventing the fusion of the 30S and 50S ribosomal subunits. It shows excellent efficacy against gram-positive bacteria, but sometimes induces immunosuppression [8]. DAP is a cyclic lipopeptide with rapid bactericidal activity against a wide range of a gram-positive bacteria, including methicillin-resistant *Staphylococcus aureus* (MRSA). However, the effectiveness and adverse reactions of these treatments for PJIs are unknown, and few reports have compared LZD and DAP. We have administered LZD since 2009 and DAP since 2014, with or without implant removal, as soon as a PJI was suspected. There were no contraindications because gram-positive bacteria caused the majority of PJIs and the antibiotic resistance was difficult to overcome. Thus, the aim of this study was to compare the effectiveness and adverse reactions of LZD and DAP as treatments for PJI. We hypothesized that there would be significantly fewer adverse events associated with DAP therapy than with LZD therapy, although the effectiveness of LZD and DAP therapy appears equivalent.

## Methods

### Study population

Between June 2009 and December 2017, 82 patients were administered LZD (Zyvox®, Pfizer Japan Inc., Tokyo, Japan) or DAP (Cubicin®, MSD K.K., Tokyo, Japan) as a treatment for a PJI at our institution. They were administered immediately following the joint aspiration for cell culture when a PJI was suspected. Some symptoms for PJIs included elevated serum C-reactive protein (CRP) and erythrocyte sedimentation rate (ESR), and clinical signs of infection (sinus tract, high fever, local heat, or swelling). LZD was used from June 2009 to July 2014, whereas DAP was used from August 2014 to December 2017. The antibiotics were not randomly selected as we conducted a retrospective study of all 82 patients (follow-up rate of 100%). The LZD group (group L, 39 patients) included 12 men and 27 women, with a mean age of 75 years (range, 37–104 years) and a mean follow-up period of 39 months (1–96 months). The infected sites were 20 hips, 18 knees, and 1 ankle. The mean duration of LZD therapy was 17 days (5–50 days) with a mean daily dose of 22.3 mg/kg/day (13.3–40.0 mg/kg/day). Surgical options used with LZD therapy included implant retention in 25 patients (no surgery in 18 and only debridement in 7), implant removal in 13 patients (subsequent arthroplasty in 13), and a shift to another appropriate antibiotic in 1 patient. The DAP group (group D, 43 patients) included 20 men and 23 women, with a mean age of 70 years (36–88 years) and a mean follow-up period of 22 months (1–48 months). The infected sites were 25 hips, 12 knees, 3 shoulders, and 3 ankles. The mean duration of DAP therapy was 24 days (1–106 days) with a mean daily dose of 6.0 mg/kg/day (3.1–10.6 mg/kg/day). Surgical options used with DAP therapy included implant retention in 24 patients (no surgery in 12 and only debridement in 12), implant removal in 13 patients (debridement and implant removal in 2 and subsequent arthroplasty in 11), and a shift to another appropriate antibiotic in 6 patients (Tables 1 and 2).

**Table 1:** Preoperative patient characteristics

Characteristics	Group L	Group D	*P* value
Number of joints	39	43	
Mean age at surgery, years (range)	75 (37–104)	70 (36–88)	0.067^a^
Gender, male to female	12:27	20:23	0.144^b^
Mean follow-up period, months (range)	45 (1–96)	22 (1–48)	*< 0.001* ^*c*^
Number of patients administered other antibiotics before this therapy	32	25	*0.019* ^*b*^
Mean dose, mg/kg/day (range)	22.3 (13.3–40.0)	6.0 (3.1–10.6)	*< 0.001* ^*c*^
Mean duration, day (range)	17 (5–50)	25 (1–106)	0.193^c^
Number of patients administered rifampicin	2	35	*0.019* ^*b*^
Surgical intervention during this therapy, number			
None	18	12	0.087^b^
Only debridement	7	12	0.286^b^
Debridement and implant removal	0	2	0.272^d^
One-stage revision	0	5	*0.035* ^*d*^
Two-stage revision	13	6	*0.038* ^*b*^
Shift to another antibiotic after identification	1	6	0.071^d^

^a^Student’s *t* test

^b^Pearson’s chi-squared test

^c^Mann-Whitney *U* test

^d^Fisher’s exact test

**Table 2:** Prosthetic joint and surgical interventions

Joints	Group L (*n* = 39)			Group D (*n* = 43)		
	Implant retention	Implant removal	Shift to another antibiotic	Implant retention	Implant removal	Shift to another antibiotic
Hip, *n*	11	8	1	10	10	5
Knee, *n*	13	5	0	9	2	1
Shoulder, *n*	0	0	0	3	0	0
Ankle, *n*	1	0	0	2	1	0

*n* number

PJIs were diagnosed according to the criteria of the Musculoskeletal Infection Society [9]. Although some patients were administered LZD or DAP prior to the identification of the pathogen by joint aspiration, a change to a more appropriate antibiotic was often made after identification. In cases where no identification of bacteria was made by joint aspiration, LZD or DAP therapy was continued if CRP levels decreased and discontinued if CRP levels did not decrease. Moreover, LZD or DAP therapy was discontinued when serious adverse events occurred. During DAP therapy, rifampicin (RFP) was added whenever possible because the synergistic effect of DAP and RFP was a promising treatment option for implant-associated MRSA infections [10, 11]. Patients who shifted to another antibiotic after identification or whose pathogens were not identified were not excluded from this study. The study was approved by our institutional review board. All patients provided informed consent for study participation and publication of findings.

### Outcome measures

After antibiotic administration, patients were followed-up at 1 week, 2 weeks, 3 weeks, 4 weeks, 8 weeks, 12 weeks, 6 months, 9 months, 1 year, and annually thereafter. Data were retrospectively analyzed by two orthopedic surgeons who were blinded to the treatment regimens. Pathogens causing the PJIs, reasons for the discontinuation of an antibiotic, and infection control rates were evaluated. Infection control was defined as a lack of clinical signs and symptoms of infection, a CRP level < 10 mg/L, an ESR < 20 mm/h, and the absence of radiological signs of infection. Therefore, a successful case was defined as not requiring implant removal due to recurrent infection after this treatment including implant retention and removal, and continuing LZD or DAP therapy without adverse events and other reasons; failure was defined as implant removal due to recurrent infection after this treatment. For the laboratory assessment, CRP levels (mg/L), red blood cell (RBC) counts (million cells/μL), platelet counts (× 10^9^/L), and estimated glomerular filtration rates (eGFR, mL/min/1.73 m^2^) were investigated.

### Statistical analysis

Two-group comparisons were conducted using Student’s *t* test or Mann-Whitney *U* test. To compare qualitative variables, Fisher’s exact test or Pearson’s chi-squared test was applied. The Wilcoxon signed-rank test was used to compare differences before and after treatment of patients. A generalized linear mixed-effects model with patients as a random effect was employed to compare the groups in terms of postoperative mean RBC count, platelet count, and eGFR. Statistical significance for a two-sided test was defined as *P* <  0.05. All analyses were performed using SAS 9.2 (SAS Institute Inc., Cary, NC, USA).

## Results

Microorganisms isolated from PJIs are shown in Table 3. The percentage of gram-positive bacteria isolated was 72% (28/39) for group L and 70% (30/43) for group D. In cases of gram-positive bacteria, the rates detected before and after LZD administration were 61% (17/28) and 39% (11/28), respectively. Moreover, the rates detected before and after DAP administration were 67% (20/30) and 33% (10/30), respectively. In cases of other microorganisms, the rates detected after LZD and DAP administration were 100% (1/1) and 100% (6/6), respectively. Reasons for the discontinuation of an antibiotic are summarized in Table 4. Clinical success rates in group D were significantly higher than those in group L (*P* <  0.05, Fisher’s exact test). The number of adverse events in group D was significantly lower than that in group L (*P* <  0.05, Fisher’s exact test). In group L, pancytopenia occurred in eight cases, and loss of appetite or nausea occurred in four cases. In group D, drug eruptions, decreased renal function, and anaphylactic shock occurred in one case each. Infection control rates are shown in Table 5. At final follow-up, among the gram-positive pathogen cases, six cases in group L and seven cases in group D required implant removal due to recurrent infection after this treatment. Whole infection control rates for gram-positive bacteria were 79% (22/28) for group L and 77% (23/30) for group D. Infection control rates for gram-positive pathogens in group L with and without implant removal were 58% and 94%, respectively, whereas those in group D with and without implant removal were 80% and 75%, respectively. In cases of methicillin-resistant bacteria, infection control rates without implant removal were higher than those with implant removal in both groups. In cases where no organisms were identified by joint aspiration, the infection control rates were 100% in both groups.

**Table 3:** Microorganisms isolated from PJIs in the current study

Pathogen	Group L, *n* (%)	Group D, *n* (%)
Gram-positive bacterium	28 (72%)	30 (70%)
Methicillin-resistant bacterium	20	13
Other gram-positive bacterium	8	17
Other microorganisms	1 (2%)	6 (14%)
No identification using joint aspiration	5 (13%)	7 (16%)
No culture	5 (13%)	0

*n* number

**Table 4:** Reasons for antibiotic discontinuation

Reasons	Group L, *n* (%)	Group D, *n* (%)	*P* value^a^
Continuation for clinical success	17 (44%)	30 (70%)	*0.025*
Clinical failure or not evaluable	3 (8%)	3 (7%)	1.000
Adverse event	12 (31%)	3 (7%)	*0.009*
Shift to another antibiotic after identification or for no efficacy	3 (8%)	6 (14%)	0.487
Fixed administration duration in advance	2 (5%)	1 (2%)	0.602
Unknown	2 (5%)	0 (0%)	0.223

*n* number

^a^Fisher’s exact test

**Table 5:** Infection control rates at final follow-up

Pathogen and surgical intervention during this therapy	Group L, % (*n*)	Group D, % (*n*)	*P* value
Gram-positive bacterium			
Whole surgical intervention	79% (22/28)	77% (23/30)	0.862^a^
Implant retention	94% (15/16)	75% (15/20)	0.147^b^
Implant removal	58% (7/12)	80% (8/10)	0.268^b^
Methicillin-resistant bacterium			
Whole surgical intervention	70% (14/20)	92% (12/13)	0.136^b^
Implant retention	89% (8/9)	100% (10/10)	0.474^b^
Implant removal	55% (6/11)	67% (2/3)	0.615^b^
No identification using joint aspiration			
Whole surgical intervention	100% (5/5)	100% (7/7)	
Implant retention	100% (5/5)	100% (3/3)	
Implant removal	(−)	100% (4/4)	

*n* number

^a^Pearson’s chi-squared test

^b^Fisher’s exact test

**Fig. 1:**
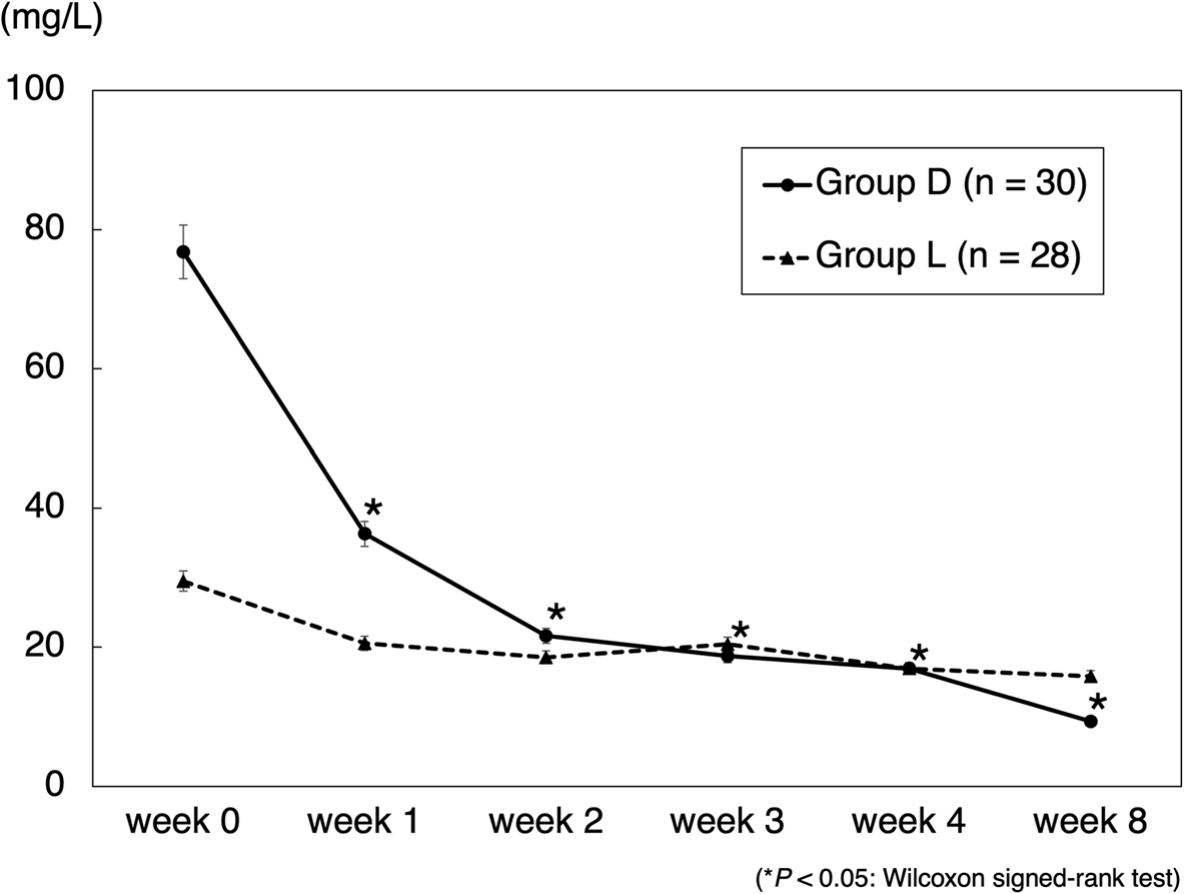
Mean CRP transition in the cases of gram-positive bacteria. Data are expressed as means and two-sided 95% confidence intervals. The Wilcoxon signed-rank test was used to compare differences before (week 0) and after the treatment of patients

**Fig. 2:**
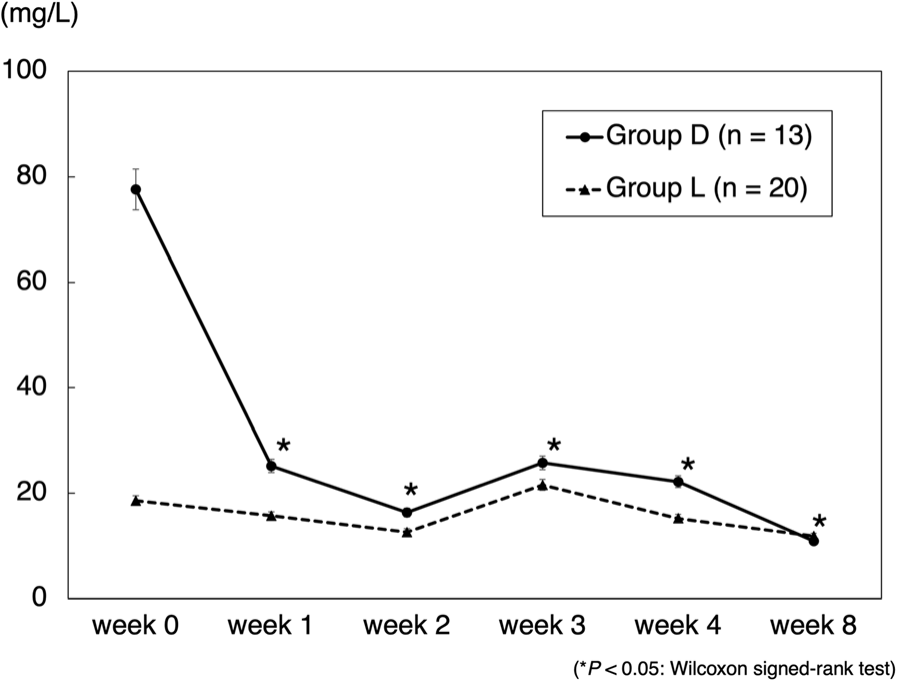
Mean CRP transition in the cases of methicillin-resistant bacteria. Data are expressed as means and two-sided 95% confidence intervals. The Wilcoxon signed-rank test was used to compare differences before (week 0) and after the treatment of patients

**Fig. 3:**
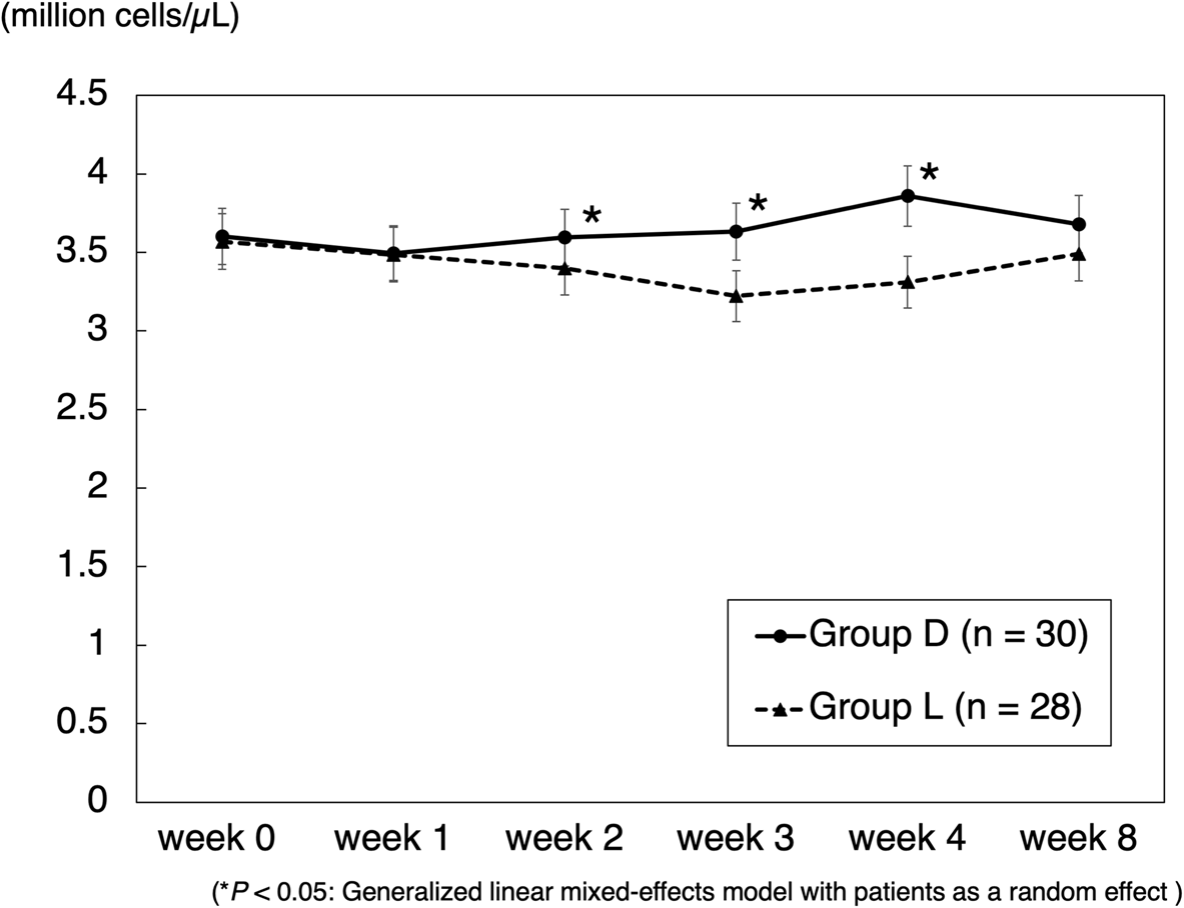
Mean red blood cell count transition. Data are expressed as means and two-sided 95% confidence intervals. A generalized linear mixed-effects model with patients as a random effect was employed to compare the groups

**Fig. 4:**
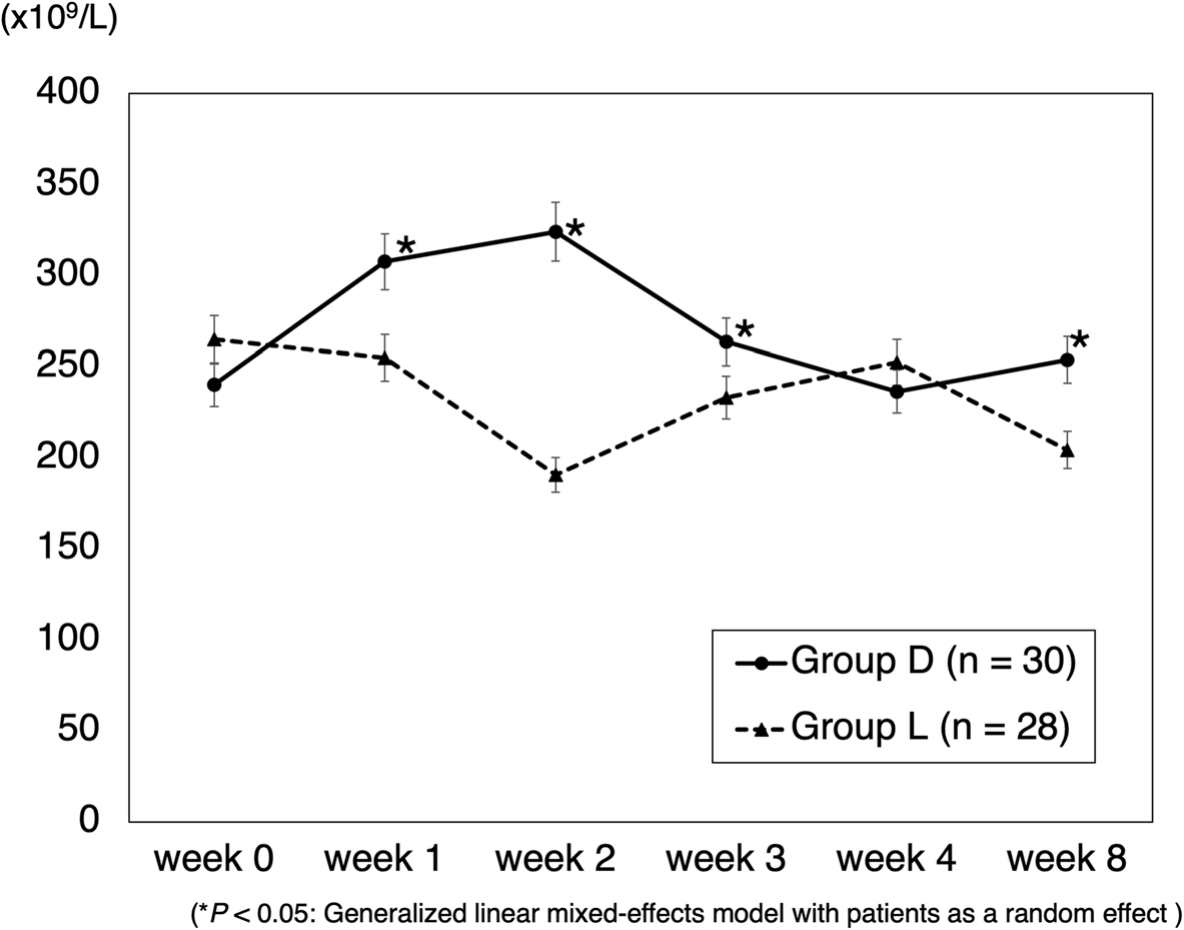
Mean platelet count transition. Data are expressed as means and two-sided 95% confidence intervals. A generalized linear mixed-effects model with patients as a random effect was employed to compare the groups

**Fig. 5:**
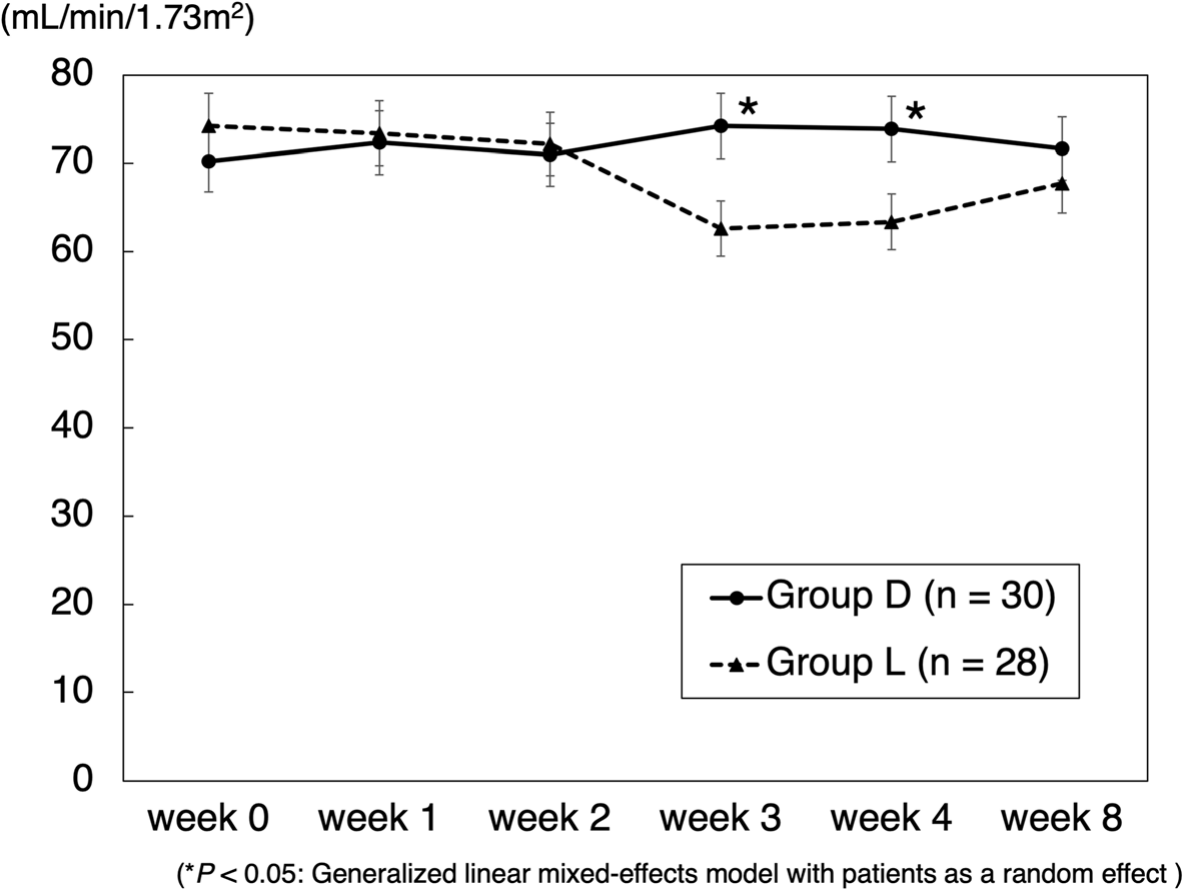
Mean eGFR transition. Data are expressed as means and two-sided 95% confidence intervals. A generalized linear mixed-effects model with patients as a random effect was employed to compare the groups

The mean CRP transition in cases of gram-positive bacteria is shown in Fig. 1. CRP levels in group D were significantly decreased. In cases where no organisms were identified by joint aspiration, a decrease in CRP levels was seen in 60% (3/5) of cases in group L and 100% (7/7) of cases in group D. The mean CRP transition in cases of methicillin-resistant bacteria is shown in Fig. 2. CRP levels in group D were significantly decreased. The mean RBC count significantly decreased in group L from weeks 1 to 2 and was significantly lower than that in group D (Fig. 3). The mean platelet count was significantly decreased in group L from weeks 0 to 1 and was significantly lower than that in group D (Fig. 4). The mean eGFR also significantly decreased in group L from weeks 2 to 3 and was significantly lower than that in group D (Fig. 5).

## Discussion

In Japan, the proportion of gram-positive PJIs is over 70%; 42% of those are MRSA [4]. In Europe and the USA, more than 50% of PJIs are typically caused by staphylococcal organisms; this trend is expected to increase [6, 7]. The increased frequency of gram-positive infections and the rise in resistance to commonly used antibiotics have led to the need for novel antibiotic therapies, such as LZD and DAP [12, 13]. According to the guidelines of the Japanese Society of Chemotherapy and the Japanese Association for Infectious Diseases, in the field of bone and joint infections, the use of either vancomycin or DAP is the first-line treatment for MRSA, with LZD or teicoplanin as alternative therapies [14]. Further, the European Registry confirmed that DAP is effective and safe in patients with osteomyelitis or those with orthopedic device infections [15, 16]. However, it is unknown which treatment (LZD or DAP) has more clinical advantages or disadvantages.

In the current study, whole infection control rates were equivalent between groups, whereas CRP levels decreased to a greater extent in group D than in group L. Furthermore, when the data were stratified by the reasons for the discontinuation of an antibiotic, group D had significantly higher clinical success rates and fewer adverse events. Morata et al. analyzed 293 PJIs from 10 articles and reported that the adverse events were frequent during prolonged administration of LZD (34.3%), requiring treatment discontinuation in 12.8% of patients [17]. Similar to our findings, adverse events were present in 31% of patients. Thus, DAP may be a safer antibiotic for the treatment of patients with PJIs. In addition, in all cases in which no organisms were identified, DAP was effective.

It is currently unclear whether implants can be retained using LZD or DAP. The European Registry demonstrated high clinical success with DAP therapy, including implant retention in 56% of patients; patients receiving both DAP and RFP showed numerically higher success rates than those who did not receive RFP concomitantly [14]. In vitro, DAP had the fastest eradication rate for MRSA embedded in a biofilm; therefore, the combination of DAP and RFP is a promising treatment option for implant-associated MRSA infections [10, 11]. Furthermore, the combination of high-dose DAP (equivalent to 8–10 mg/kg/day in humans) and RFP was highly effective for the treatment of foreign body-related MRSA infections [18, 19]. Lora-Tamayo et al. also analyzed 18 staphylococcal PJIs in a multicenter study and concluded that high-dose DAP (10 mg/kg/day) plus RFP was a good initial treatment for PJIs with implant retention [20]. RFP is an important addition to DAP for the treatment of PJIs, with or without implant removal. In the current study, however, the mean daily dose of DAP for successful cases of implant retention was not always high; hence, the necessity for high-dose DAP remains controversial. On the other hand, some authors showed good clinical results using LZD as a treatment for PJIs, including those with implant retention [17, 21–26]. However, Tornero et al. analyzed the results of 143 PJIs treated with debridement and implant retention and concluded that the selection and duration of the co-administered antibiotics were important [26]. Although the free concentrations of LZD to treat soft tissues and bones infected with MRSA or other gram-positive bacteria are higher than those of DAP, DAP may be a better treatment for PJI because of its high penetration into biofilms, low bactericidal concentration, and synergistic effects with RFP [10, 11, 27–29]. In addition, the decrease in CRP levels in cases in which no organisms were identified by joint aspiration was seen in 60% of group L patients and 100% of group D patients; infection control rates were 100% for both groups. Therefore, if a PJI is suspected, we recommend the immediate initiation of DAP, even before the precise identification of the pathogen.

Some limitations of our study must be noted. First, the sample size was small, involving only 82 individuals. Furthermore, the current study was not performed as a randomized controlled trial. Patients with refractory PJIs required treatment on a case-by-case basis for the optimal surgical intervention. The use of RFP may also overestimate the actual effect of DAP. Additionally, the status of a microbial infection is largely dependent on the timing of follow-up cultures, which may vary based on individual patients and practitioners. Second, some patients who did not undergo implant removal after treatment continued to take other antibiotics orally, suggesting the existence of recurrent infections. Third, in the current study, the percentage of patients with gram-positive bacteria was 72% for group L and 70% for group D. We administered antibiotics when a PJI was suspected, even before the identification of the specific pathogen, because the majority of PJIs are caused by gram-positive bacteria and antibiotic resistance is difficult to overcome. Such an approach may lead to excessive levels of an antibiotic being administered, but treatments can be switched to a more appropriate antibiotic after pathogen identification. There was the possibility of misdiagnosis even though the transition of laboratory results indicated a PJI.

## Conclusion

For refractory PJIs, the use of LZD or DAP was beneficial for the treatment of MRSA and other gram-positive pathogens, and the effectiveness of both LZD and DAP was equivalent in terms of their infection control rates. Some patients did not require implant removal during LZD and DAP therapies. Furthermore, in cases where no pathogen was identified, the decrease in CRP levels was seen in 60% of patients in group L and in 100% of patients in group D. However, after DAP therapy, CRP levels were significantly decreased and adverse events were significantly fewer than in patients treated with LZD.

## Data Availability

All data generated or analyzed during this study are included in this published article.

## Abbreviations

CRP: C-reactive protein
DAP: Daptomycin
eGFR: Estimated glomerular filtration rates
ESR: Erythrocyte sedimentation rate
LZD: Linezolid
MRSA: Methicillin-resistant *Staphylococcus aureus*
PJI: Periprosthetic joint infection
RBC: Red blood cell
RFP: Rifampicin

## References

[1] Dale H, Fenstad AM, Hallan G, Havelin LI, Furnes O, Overgaard S (2012). Increasing risk of prosthetic joint infection after total hip arthroplasty. Acta Orthop.

[2] Ueda N, Oe K, Nakamura T, Tsuta K, Iida H, Saito T (2019). Sonication of extracted implants improves microbial detection in patients with orthopedic implant-associated infections. J Arthroplast.

[3] Osmon DR, Berbari EF, Berendt AR, Lew D, Zimmerli W, Steckelberg JM (2013). Infectious Diseases Society of America. Diagnosis and management of prosthetic joint infection: clinical practice guidelines by the Infectious Diseases Society of America. Clin Infect Dis.

[4] Yamamoto K, Masaoka T, Ishii Y, Iida H, Matsuno T, Satomi K (2010). Epidemiology of surgical site infection following implant surgery. Orthop Surg Traumatol.

[5] Tande AJ, Patel R (2014). Prosthetic joint infection. Clin Microbiol Rev.

[6] Aggarwal VK, Bakhshi H, Ecker NU, Parvizi J, Gehrke T, Kendoff D (2014). Organism profile in periprosthetic joint infection: pathogens differ at two arthroplasty infectionreferral centers in Europe and in the United States. J Knee Surg.

[7] Kapadia BH, Berg RA, Daley JA, Fritz J, Bhave A, Mont MA (2016). Periprosthetic joint infection. Lancet..

[8] French G (2003). Safety and tolerability of linezolid. J Antimicrob Chemother.

[9] Parvizi J, Zmistowski B, Berbari EF, Bauer TW, Springer BD, Della Valle CJ (2011). New definition for periprosthetic joint infection: from the Workgroup of the Musculoskeletal Infection Society. Clin Orthop Relat Res.

[10] Raad I, Hanna H, Jiang Y, Dvorak T, Reitzel R, Chaiban G (2007). Comparative activities of daptomycin, linezolid, and tigecycline against catheter-related methicillin-resistant Staphylococcus bacteremic isolates embedded in biofilm. Antimicrob Agents Chemother.

[11] John AK, Baldoni D, Haschke M, Rentsch K, Schaerli P, Zimmerli W (2009). Efficacy of daptomycin in implant-associated infection due to methicillin-resistant Staphylococcus aureus: importance of combination with rifampin. Antimicrob Agents Chemother.

[12] Tiemersma EW, Bronzwaer SL, Lyytikäinen O, Degener JE, Schrijnemakers P, Bruinsma N (2004). Methicillin-resistant Staphylococcus aureus in Europe, 1999–2002. Emerg Infect Dis.

[13] Deresinski S (2007). Counterpoint: vancomycin and Staphylococcus aureus--an antibiotic enters obsolescence. Clin Infect Dis.

[14] Niki Y (2017). Practical guidelines for the management and treatment of infections caused by MRSA.

[15] Gonzalez-Ruiz A, Gargalianos-Kakolyris P, Timerman A, Sarma J, José González Ramallo V (2015). Daptomycin in the clinical setting: 8-year experience with gram-positive bacterial infections from the EU-CORE(SM) registry. Adv Ther.

[16] Malizos K, Sarma J, Seaton RA, Militz M, Menichetti F, Riccio G (2016). Daptomycin for the treatment of osteomyelitis and orthopaedic device infections: real-world clinical experience from a European registry. Eur J Clin Microbiol Infect Dis.

[17] Morata L, Tornero E, Martínez-Pastor JC, García-Ramiro S, Mensa J, Soriano A (2014). Clinical experience with linezolid for the treatment of orthopaedic implant infections. J Antimicrob Chemother.

[18] Garrigós C, Murillo O, Euba G, Verdaguer R, Tubau F, Cabellos C (2010). Efficacy of usual and high doses of daptomycin in combination with rifampin versus alternative therapies in experimental foreign-body infection by methicillin-resistant Staphylococcus aureus. Antimicrob Agents Chemother.

[19] Saleh-Mghir A, Muller-Serieys C, Dinh A, Massias L, Crémieux AC (2011). Adjunctive rifampin is crucial to optimizing daptomycin efficacy against rabbit prosthetic joint infection due to methicillin-resistant Staphylococcus aureus. Antimicrob Agents Chemother.

[20] Lora-Tamayo J, Parra-Ruiz J, Rodríguez-Pardo D, Barberán J, Ribera A, Tornero E (2014). High doses of daptomycin (10 mg/kg/d) plus rifampin for the treatment of staphylococcal prosthetic joint infection managed with implant retention: a comparative study. Diagn Microbiol Infect Dis.

[21] Bassetti M, Di Biagio A, Cenderello G, Del Bono V, Palermo A, Cruciani M (2001). Linezolid treatment of prosthetic hip infections due to methicillin-resistant Staphylococcus aureus (MRSA). J Inf Secur.

[22] Lovering AM, Zhang J, Bannister GC, Lankester BJ, Brown JH, Narendra G (2002). Penetration of linezolid into bone, fat, muscle and haematoma of patients undergoing routine hip replacement. J Antimicrob Chemother.

[23] Rana B, Butcher I, Grigoris P, Murnaghan C, Seaton RA, Tobin CM (2002). Linezolid penetration into osteo-articular tissues. J Antimicrob Chemother.

[24] Vercillo M, Patzakis MJ, Holtom P, Zalavras CG (2007). Linezolid in the treatment of implant-related chronic osteomyelitis. Clin Orthop Relat Res.

[25] Morata L, Senneville E, Bernard L, Nguyen S, Buzelé R, Druon J (2014). A retrospective review of the clinical experience of linezolid with or without rifampicin in prosthetic joint infections treated with debridement and implant retention. Infect Dis Ther.

[26] Tornero E, Morata L, Martínez-Pastor JC, Angulo S, Combalia A, Bori G (2016). Importance of selection and duration of antibiotic regimen in prosthetic joint infections treated with debridement and implant retention. J Antimicrob Chemother.

[27] Traunmüller F, Schintler MV, Spendel S, Popovic M, Mauric O, Scharnagl E (2010). Linezolid concentrations in infected soft tissue and bone following repetitive doses in diabetic patients with bacterial foot infections. Int J Antimicrob Agents.

[28] Traunmüller F, Schintler MV, Metzler J, Spendel S, Mauric O, Popovic M (2010). Soft tissue and bone penetration abilities of daptomycin in diabetic patients with bacterial foot infections. J Antimicrob Chemother.

[29] Huang YT, Liao CH, Teng LJ, Hsueh PR (2008). Comparative bactericidal activities of daptomycin, glycopeptides, linezolid and tigecycline against blood isolates of Gram-positive bacteria in Taiwan. Clin Microbiol Infect.

